# GSTM1 Copy Number and Kidney Disease in People With HIV

**DOI:** 10.1016/j.ekir.2022.05.003

**Published:** 2022-05-13

**Authors:** Rachel K.Y. Hung, Kerry-Lee Rosenberg, Victor David, Elizabeth Binns-Roemer, John W. Booth, Rachel Hilton, Julie Fox, Fiona Burns, Andrew Ustianowski, Catherine Cosgrove, Lisa Hamzah, James E. Burns, Amanda Clarke, David Chadwick, David A. Price, Stephen Kegg, Lucy Campbell, Kate Bramham, Caroline A. Sabin, Frank A. Post, Cheryl A. Winkler, GEN-AFRICA Study Group, Anele Waters, James Hand, Chris Clarke, Sarah Murphy, Maurice Murphy, Marion Campbell, Celia Richardson, Alyson Knott, Gemma Weir, Rebecca Cleig, Helena Soviarova, Lisa Barbour, Tanya Adams, Vicky Kennard, Vittorio Trevitt, Rachael Jones, Jeremy Levy, Alexandra Schoolmeester, Serah Duro, May Rabuya, Deborah Jordan, Teresa Solano, Hiromi Uzu, Karen Williams, Julianne Lwanga, Linda Ekaette Reid-Amoruso, Hannah Gamlen, Robert J. Stocker, Fiona Ryan, Anele Waters, Karina Mahiouz, Tess Cheetham, Claire Williams, Achyuta Nori, Caroline Thomas, Sivaraj Venkateshwaran, Jessica Doctor, Andrea Berlanga, Beatriz Santana-Suarez, Leigh McQueen, Priya Bhagwandin, Bee Barbini, Emily Wandolo, Tim Appleby, Deborah Jordan, Lois Driver, Sophy Parr, Hongbo Deng, Julie Barber, Andrew Crowe, Chris Taylor, Mary Poulton, Vida Boateng, Marie-Pierre Klein, Caitlin O'Brien, Samuel Ohene-Adomako, Christian Buckingham, Daniel Trotman, Killian Quinn, Kate Flanagan, Verity Sullivan, Holly Middleditch, Itty Samuel, Elizabeth Hamlyn, Candice McDonald, Ana Canoso, Emeka Agbasi, Maria Liskova, Sarah Barber, Amanda Samarawickrama, Zoe Ottaway, Claire Norcross, Amelia Oliveira, Jane Minton, Gary Lamont, Ruby Cross, Gaushiya Saiyad, Shadia Ahmed, Rebecca Ashworth, Nicola Window, J. Murira, Khine Phyu, Gabriella Lindergard, Jonathan Shaw, Sarah Holland, Claire Fox, Jan Flaherty, Margaret-Anne Bevan, Valerie George, Marie Branch, Pauline Lambert, Adele Craggs, Sarah Pett, Hinal Lukha, Nina Vora, Marzia Fiorino, Maria Muller Nunez, Deirdre Sally, Erica Pool, Rebecca Matthews, Tara Stothard, Bijal Patel, Ian McVittie, Ciara Kennedy, Uli Shwab, Brendan Payne, Sarah Duncan, Jill Dixon, Mathias Schmid, Adam Evans, Christopher Duncan, Ewan Hunter, Yusri Taha, Natasha Astill, Jonathan Ainsworth, Rachel Vincent, Chloe Saad, Sarah Skinner, Hocine Azzoug, Judith Russell, Tarik Moussaoui, Celia Richardson, Emily Mabonga, Donna Ward, J. Francoise, W. Larbi, Sue Mitchell, A. Manning, V. Russell, Mark Harber, Nnenna Ngwu, Jonathan Edwards, Nargis Hemat, Tom Fernandez, Filippo Ferro, Jorge Ferreira, Alice Nightingale, Tasha Oakes-Monger, Darwin Matila, Pedro Nogueira, Victoria Mutagwanya, Catherine Emily Isitt, Helen Webb, Joyce Popoola, Kate Korley, Mark Mencias, Patricia Ribeiro, Rajeshwar Ramkhelawn, Sandra Oliva Lara, Sara Sajijad, Alan Winston, Jeremy Levy, Amber Shaw, Claire Petersen, Kyle Ring, Melanie Rosenvinge, Chloe Saad, Sarah Skinner, Thembi Moyo, Faith Odong, Katherine Gantert, Tina Ibe, Denis Onyango, Teresa Hill

**Affiliations:** 1 King’s College London, London, UK; 2 Royal Free London Hospital NHS Foundation Trust, London, UK; 3 Basic Research Laboratory, Frederick National Laboratory for Cancer Research and the National Cancer Institute, Frederick, USA; 4 Barts Health NHS Trust, London, UK; 5 Guy’s and St Thomas’ NHS Foundation Trust, London, UK; 6 Pennine Acute Hospitals NHS Foundation Trust, Manchester, UK; 7 St George’s Hospital NHS Foundation Trust, London, UK; 8 University College London, London, UK; 9 Central and North West London NHS Foundation Trust, London, UK; 10 Brighton and Sussex University Hospital NHS Trust, Brighton, UK; 11 Brighton and Sussex Medical School Department of Infectious Disease, Brighton, UK; 12 South Tees Hospitals NHS Foundation Trust, Middlesbrough, UK; 13 The Newcastle Upon Tyne Hospitals, Newcastle, UK; 14 Lewisham and Greenwich NHS Trust, London, UK; 15 King’s College Hospital NHS Foundation Trust, London, UK

**Keywords:** Africa, APOL1, GSTM1, HIV, kidney, oxidative stress

Oxidative stress has been implicated in the pathogenesis and progression of chronic kidney disease (CKD). An imbalance between increased production of reactive oxygen species and reduced antioxidant defenses results in disruption to downstream cellular signaling and subsequent renal cell apoptosis and senescence, fibrosis, and vascular injury.^1^ Genetic variants that improve the capacity to mitigate oxidative stress may therefore be protective against the development of CKD.

The glutathione-S-transferases play a role in the conjugation of prooxidant species with glutathione to facilitate the elimination of reactive oxygen species. *GSTM1* is the gene encoding one such isoenzyme. This gene copy number has undergone gene deletion and expansion so chromosomes have no copies, 1 copy or, in rare cases, 2 copies of the gene. Two copies of the active allele are required for enzymatic activity (haploinsufficiency); those homozygous for the null allele, *GSTM1*(0), completely lack enzyme production. Individuals with the inactive *GSTM1* genotypes (*GSTM1* 0/0 or 1/0) have been found to be at higher risk of common malignancies, atherosclerosis, coronary heart disease, and CKD progression.S1^,^S2 This study sought to investigate the relationship between *GSTM1* genotype and prevalent CKD and the interaction between *GSTM1* and *APOL1* carrier status,^2^, ^3^, ^4^ in a cohort of Black people with HIV in the United Kingdom.^5^^,^^6^

Characteristics of the 2762 participants are summarized in Supplementary Table S1. Of these, 2075 (75.1%) had *GSTM1* inactive genotypes whereas 687 (24.9%) carried 2 or 3 copies (active genotypes). The mean age of the participants was 48 years, and 57% were female. Most participants were established on antiretroviral treatment with suppressed HIV RNA levels; HIV parameters, hepatitis coinfection status, and prevalence of hypertension, diabetes, and cardiovascular disease did not differ by *GSTM1* status. Kidney function (estimated glomerular filtration rate [eGFR]) and the prevalence of *APOL1* risk variants and sickle cell trait were similar for the 2 *GSTM1* groups (Figure 1a–c and Supplementary Figure S1).

**Figure 1:**
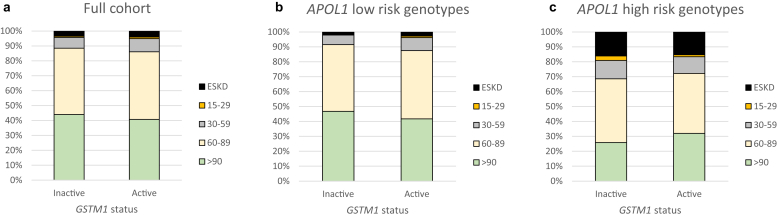
Distribution of eGFR in participants stratified by *GSTM1* genotype, overall (a) and in those with *APOL1* low-risk (b) and high-risk (c) genotypes.

In the overall study population, *GSTM1* inactive genotypes were not associated with an increased risk of kidney disease (eGFR <60 or <90 ml/min per 1.73 m^2^ or stage 5 CKD), whereas these genotypes were associated with reduced odds of albuminuria (Table 1). There was no significant interaction between *GSTM1* genotype and *APOL1* status for most kidney outcomes. When participants were stratified by *APOL1* status (Supplementary Table S2), *GSTM1* inactive genotypes in those with *APOL1* low-risk genotypes were associated with reduced odds of eGFR <60 ml/min per 1.73 m^2^ (odds ratio 0.65 [95% CI 0.49–0.87]) and albuminuria (odds ratio 0.77 [0.61–0.99]). In those with *APOL1* high-risk genotypes, *GSTM1* inactive genotypes were not associated with eGFR <60 and <90 ml/min per 1.73 m^2^ or stage 5 CKD.

**Table 1:** Associations between *GSTM1* status (inactive vs. active) and renal outcomes, overall and stratified by *APOL1* status

Kidney outcomes	Stratification by APOL1 status	OR	95% CI	*P* value	Interaction between *APOL1* and *GSTM1* genotypes
Stage 5 CKD	All	0.86	0.55–1.34	0.50	0.19
	APOL1 HRG	1.06	0.52–2.19	0.87	
	APOL1 LRG	0.57	0.31–1.05	0.07	
eGFR <60 ml/min per 1.73 m^2^	All	0.81	0.62–1.04	0.10	0.07
	APOL1 HRG	1.19	0.67–2.12	0.55	
	APOL1 LRG	0.65	0.49–0.87	0.004	
eGFR <90 ml/min per 1.73 m^2^	All	0.87	0.73–1.04	0.13	0.10
	APOL1 HRG	1.35	0.76–2.37	0.30	
	APOL1 LRG	0.81	0.68–0.98	0.03	
uACR >3 mg/mmol	All	0.78	0.63–0.98	0.04	0.82
	APOL1 HRG	0.82	0.43–1.56	0.55	
	APOL1 LRG	0.77	0.61–0.99	0.04	
uPCR >50 mg/mmol	All	0.81	0.55–1.18	0.27	0.04
	APOL1 HRG	0.66	0.24–1.77	0.41	
	APOL1 LRG	0.84	0.55–1.27	0.40	

CKD, chronic kidney disease; eGFR, estimated glomerular filtration rate; HRG, high-risk genotype (G1/G1, G1/G2, G2/G2); LRG, low-risk genotype (G0/G0, G1/G0, G2/G0); OR, odds ratio; uACR, urine albumin/creatinine ratio; uPCR, urine protein/creatinine ratio.

The inactive *GSTM1* genotype was defined by carriage of the *GSTM1*(0) null allele (i.e., *GSTM1*[1/0] and *GSTM1*[0/0]); the *GSTM1* active group is homozygous for the active allele (*GSTM1*[1/1]). Results from univariable logistic regression analysis.

In contrast to some existing evidence in Black populations with impaired kidney function,^3^^,^^4^ and consistent with recent data in people with HIV from the Eastern Congo,^7^ we found no evidence for an increased risk of kidney disease in individuals with *GSTM1* inactive genotypes. In addition, we found no evidence that *GSTM1* inactive genotypes amplify the deleterious effect of the *APOL1* high-risk genotypes.

Data from the African American Study of Kidney Disease and Hypertension revealed an association between *GSTM1* inactive genotypes and accelerated progression of CKD in a cohort of 692 Black Americans with hypertensive kidney disease, with worse progression in *APOL1* high-risk genotypes.^3^ Our cohort is substantially larger than those included in the African American Study of Kidney analyses and differs in that only 32% (as compared with all participants in African American Study of Kidney) had a diagnosis of hypertension, and that most of our participants had normal kidney function. It is possible that GSTM1 loss is implicated in the pathogenesis of hypertensive renal disease but is less significant in other or HIV-associated pathologies. Alternatively, as oxidative stress is increased in CKD,^8^ GSTM1 loss may have had a larger impact on kidney disease progression in the African American Study of Kidney study. It is possible that the potential protective effect of GSTM1 becomes important in declining eGFR and that this association was not captured in our cross-sectional study.

Evidence from the Atherosclerosis Risk in Communities Study revealed a 66% increased risk of kidney failure in both Black and White individuals with *GSTM1* inactive genotypes, compared with those with active genotypes.^4^ This study included 2254 Black participants with largely normal kidney function (mean eGFR 112 ml/min per 1.73 m^2^). The increased risk persisted after adjustment for clinical risk factors, including diabetes and hypertension. No significant association was identified, however, between *GSTM1* allele status and incident CKD. There is evidence to suggest that the protective, antioxidant effects of *GSTM1* are of greater importance in a uremic environment (i.e., at lower GFR), and this may account for the disparity between risk of incident CKD and kidney failure in this cohort.^8^ However, a large study by Zhang *et al.*^9^ also failed to reveal an association between GSTM1 loss and kidney failure in either Black (*n =* 796) or White participants (*n =* 46,187).

Our study comprises the largest cohort of Black participants in which the association between *GSTM1* status and CKD has been explored; the *GSTM1* groups were indistinguishable in terms of HIV parameters and relevant comorbidities, such as hypertension and diabetes, and *APOL1* renal risk status. This is also the largest study in which the association between *GSTM1* status and kidney outcomes stratified by *APOL1* genotype has been evaluated. Limitations include its cross-sectional study design, the positive HIV status of all participants which may preclude extrapolation to non-HIV populations, and the modest numbers of participants with the *GSTM1* active genotypes and high-risk *APOL1* genotypes, which may have rendered the study underpowered to detect an interaction between deleterious kidney outcomes and *APOL1* carrier status. In summary, this cross-sectional study does not support some earlier observations that *GSTM1* inactive genotype is a risk factor for kidney disease in Black individuals. Furthermore, *GSTM1* inactive genotypes in this population do not seem to amplify the deleterious effects of the high-risk *APOL1* genotype. Further studies in people with HIV are required to investigate the role of *GSTM1* inactive genotypes in CKD progression among those with advanced kidney disease and proteinuria.

## Appendix

### List of GEN-AFRICA Group

Genetic markers of chronic kidney disease in people of African ancestry with HIV (GEN-AFRICA) Study Group Barts Health NHS Trust, London (John Booth [PI], Anele Waters, James Hand, Chris Clarke, Sarah Murphy, Maurice Murphy); Brighton and Sussex University Hospitals, Brighton (Marion Campbell, Amanda Clarke [PI], Celia Richardson, Alyson Knott, Gemma Weir, Rebecca Cleig, Helena Soviarova, Lisa Barbour, Tanya Adams, Vicky Kennard, Vittorio Trevitt); Chelsea and Westminster Hospital, London (Rachael Jones [PI], Jeremy Levy, Alexandra Schoolmeester, Serah Duro; Guy’s and St Thomas’ Hospital, London (Rachel Hilton [PI], Julie Fox, May Rabuya, Lisa Hamzah, Deborah Jordan, Teresa Solano, Hiromi Uzu, Karen Williams, Julianne Lwanga, Linda Ekaette Reid-Amoruso, Hannah Gamlen, Robert J. Stocker, Fiona Ryan, Anele Waters, Karina Mahiouz, Tess Cheetham, Claire Williams, Achyuta Nori, Caroline Thomas, Sivaraj Venkateshwaran, Jessica Doctor, Andrea Berlanga); King’s College Hospital, London (Frank Post [CI], Beatriz Santana-Suarez, Leigh McQueen, Priya Bhagwandin, Lucy Campbell, Bee Barbini, Emily Wandolo, Tim Appleby, Deborah Jordan, Lois Driver, Sophy Parr, Hongbo Deng, Julie Barber, Andrew Crowe, Chris Taylor, Mary Poulton, Vida Boateng, Marie-Pierre Klein, Caitlin O'Brien, Samuel Ohene-Adomako, Christian Buckingham, Daniel Trotman, Killian Quinn, Kate Flanagan, Verity Sullivan, Holly Middleditch, Itty Samuel, Elizabeth Hamlyn, Candice McDonald, Ana Canoso, Emeka Agbasi, Maria Liskova, Sarah Barber, Amanda Samarawickrama, Zoe Ottaway, Claire Norcross, Amelia Oliveira, Kate Bramham); Leeds Teaching Hospitals NHS Trust, Leeds (Jane Minton [PI], Gary Lamont, Ruby Cross, Gaushiya Saiyad, Shadia Ahmed, Rebecca Ashworth, Nicola Window, J Murira, Khine Phyu); North Manchester General Hospital, Manchester (Andrew Ustianowski [PI], Gabriella Lindergard, Jonathan Shaw, Sarah Holland, Claire Fox, Jan Flaherty, Margaret-Anne Bevan, Valerie George); South Tees Hospitals NHS Foundation Trust, Middlesbrough (David Chadwick [PI], Marie Branch, Pauline Lambert, Adele Craggs); Mortimer Market Centre, Central and NorthWest London NHS Foundation Trust, London (Sarah Pett [PI], Hinal Lukha, Nina Vora, Marzia Fiorino, Maria Muller Nunez, Deirdre Sally, James E. Burns, Erica Pool, Rebecca Matthews); Newcastle upon Tyne Hospitals, Newcastle (David Ashley Price [PI], Tara Stothard, Bijal Patel, Ian McVittie, Ciara Kennedy, Uli Shwab, Brendan Payne, Sarah Duncan, Jill Dixon, Mathias Schmid, Adam Evans, Christopher Duncan, Ewan Hunter, Yusri Taha, Natasha Astill); National Cancer Institute, Frederick, USA (Cheryl Winkler, Elizabeth Binns-Roemer, Victor David); North Middlesex University Hospital, London (Jonathan Ainsworth, Rachel Vincent [PI]); Queen Elizabeth Hospital, Woolwich (Stephen Kegg [PI], Chloe Saad, Sarah Skinner, Hocine Azzoug, Judith Russell, Tarik Moussaoui, Celia Richardson, Emily Mabonga, Donna Ward, J. Francoise, W. Larbi, Sue Mitchell, A. Manning, V. Russell); Royal Free London Hospital, London (Fiona Burns [PI], Mark Harber, Nnenna Ngwu, Jonathan Edwards, Nargis Hemat, Tom Fernandez, Filippo Ferro, Jorge Ferreira, Alice Nightingale, Tasha Oakes-Monger, Darwin Matila, Pedro Nogueira, Victoria Mutagwanya); St. Georges University Hospitals, London (Catherine Cosgrove [PI], Lisa Hamzah, Catherine Emily Isitt, Helen Webb, Joyce Popoola, Kate Korley, Mark Mencias, Patricia Ribeiro, Rajeshwar Ramkhelawn, Sandra Oliva Lara, Sara Sajijad); Imperial College Healthcare NHS Trust, London (Alan Winston [PI], Jeremy Levy, Amber Shaw, Claire Petersen, Kyle Ring); University Hospital Lewisham, London (Melanie Rosenvinge [PI], Chloe Saad, Sarah Skinner, Thembi Moyo, Faith Odong, Katherine Gantert, Tina Ibe); Africa Advocacy Foundation (Denis Onyango); UK CHIC cohort (Caroline Sabin [PI], Teresa Hill).

## Supplementary Material

**Supplementary Methods.**

**Supplementary References.**

**Figure S1.** Distribution of eGFR in participants stratified by *GSTM1* copy number, overall (a) and in those with *APOL1* low-risk (b) and high-risk (c) genotypes.

**Table S1.** Characteristics of study participants stratified by *GSTM1* status.

**Table S2.** Characteristics of study participants stratified by *APOL1* and *GSTM1* status.
