# Supplementary material for: GSTM1 Copy Number and Kidney Disease in People With HIV
Source: Kidney Int Rep. 2022 May 13;7(8):1901–4. doi: 10.1016/j.ekir.2022.05.003 (PMC9366293; doi:10.1016/j.ekir.2022.05.003)
Supplement: Supplementary File (PDF) [file mmc1.pdf]

Methods

DNA was extracted from buffy coats using Qiagen extraction kits. *GSTM1* gene copy number was estimated using a TaqMan Copy Number Assay (ThermoFisher, Waltham, MA; Hs02575461_cn). Real time PCR were performed in a 5 µL reaction using manufacturer's protocol with RNAse P serving as an internal control. Samples were run in quadruplicate on an Applied Biosystems 7900-HT real-time instrument and scored as 0, 1 or 2 gene copies using Applied Biosystems Copycaller software (version 2.1). Only samples that worked 3 or more times and were always concordant were scored. The inactive *GSTM1* genotype was defined by carriage of the *GSTM1(0)* null allele [i.e. *GSTM1(1/0)* and *GSTM1(0/0)*] while the *GSTM1* active group, by definition, is homozygous for the active allele [*GSTM1(1/1)*]. *APOL1* variant sites were genotyped using custom TaqMan assays (ThermoFisher Scientific, Waltham, MA, USA): rs73885319 (Assay ID-AH20SD1), rs60910145 (Assay ID-AHWR1JA), and rs71785313 (Assay ID-AH1RT7T). The *APOL1* high–risk group was defined by carriage of any two copies of the G1 or G2 haplotypes (i.e. G1/G1, G1/G2, or G2/G2 genotypes.

Participants were divided into *GSTM1* inactive and *GSTM1* active groups. Logistic regression was used to describe the association between *GSTM1* inactive genotypes and kidney disease (eGFR <60 and <90 mL/min/1.73m^2, stage 5 CKD, proteinuria (uPCR >50 mg/mmol) and albuminuria (ACR >3 mg/mmol); likelihood ratio tests were used to assess the strength of association at each level. All statistical analyses were done using STATA v16 (StataCorp, College Station, Tx).

Supplementary references

S1. Carlsten C, Sagoo GS, Frodsham AJ*, et al.* Glutathione S-transferase M1 (GSTM1) polymorphisms and lung cancer: a literature-based systematic HuGE review and meta-analysis. *American journal of epidemiology* 2008; **167:** 759-774.

S2. Yang M, Zhao J, Xing L*, et al.* Association between GSTM1 null genotype and coronary artery disease risk: a meta-analysis. *Med Sci Monit* 2014; **20:** 1550-1555.

Figure S1 Distribution of eGFR in participants stratified by *GSTM1* copy number, overall (A) and in those with *APOL1* low-risk (B) and high-risk (C) genotypes

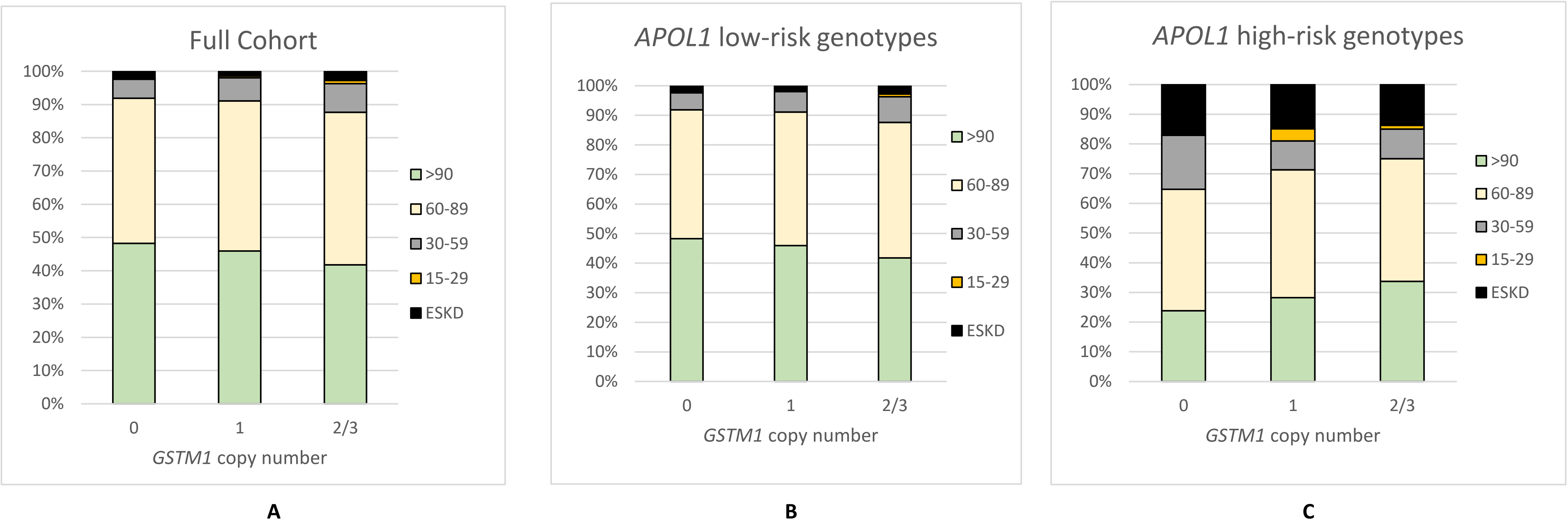

Table S1 **Characteristics of study participants stratified by *GSTM1* status**

			GSTM1 status		
		**Total**	**Inactive**	**Active**	**p-value**
		N=2,762	N=2,075	N=687	
Age, years	mean (sd)	48.0 (10.3)	48.0 (10.3)	48.0 (10.2)	0.92
Sex, female	n (%)	1,574 (57.0)	1,190 (57.4)	384 (55.9)	0.5
Region of ancestry					<0.001
East Africa	n (%)	512 (18.5)	430 (20.7)	82 (11.9)	
South Africa	n (%)	735 (26.6)	528 (25.4)	207 (30.1)	
Central Africa	n (%)	154 (5.6)	113 (5.4)	41 (6.0)	
West Africa	n (%)	844 (30.6)	617 (29.7)	227 (33.0)	
Caribbean	n (%)	342 (12.4)	250 (12.0)	92 (13.4)	
Other	n (%)	175 (6.3)	137 (6.6)	38 (5.5)	
HIV mode of acquisition					0.42
Heterosexual	n (%)	2,254 (81.6)	1,686 (81.3)	568 (82.7)	
MSM	n (%)	70 (2.5)	59 (2.8)	11 (1.6)	
Vertical	n (%)	230 (8.3)	174 (8.4)	56 (8.2)	
Blood products	n (%)	22 (0.8)	18 (0.9)	4 (0.6)	
Unknown	n (%)	186 (6.7)	138 (6.7)	48 (7.0)	
Time since HIV diagnosis, years	mean (sd)	13.9 (6.5)	13.9 (6.5)	14.1 (6.2)	0.42
Previous AIDS	n (%)	643 (24.0)	475 (23.7)	168 (25.1)	0.45
Nadir CD4 cell count, cells/mm^3	median (IQR)	200 (80-337)	202 (85-337)	197 (67-341)	0.27
Recent CD4 cell count, cells/mm^3	median (IQR)	560 (399-735)	561.0 (397-730)	558 (407-755)	0.80
On antiretroviral therapy	n (%)	2,731 (98.9)	2,048 (98.7)	683 (99.4)	0.12
HIV RNA <200 copies/mL	n (%)	2,572 (93.1)	1,943 (93.6)	629 (91.6)	0.06
HBsAg positive	n (%)	156 (5.7)	112 (5.5)	44 (6.4)	0.34
Anti-HCV positive	n (%)	37 (1.4)	32 (1.6)	5 (0.7)	0.11
Diabetes	n (%)	273 (10.0)	200 (9.7)	73 (10.7)	0.46
Hypertension	n (%)	884 (32.0)	664 (32.0)	220 (32.0)	0.99
Cardiovascular disease	n (%)	121 (4.4)	93 (4.5)	28 (4.1)	0.65

BMI, kg/m^2					0.59
<18.5	n (%)	22 (0.8)	16 (0.8)	6 (0.9)	
18.5-24.9	n (%)	610 (22.5)	471 (23.1)	139 (20.6)	
25-29.9	n (%)	982 (36.1)	734 (36.0)	248 (36.7)	
≥30	n (%)	1,103 (40.6)	820 (40.2)	283 (41.9)	
Smoking status					0.16
Never	n (%)	2,133 (77.2)	1,586 (76.4)	547 (79.6)	
Ex	n (%)	308 (11.2)	235 (11.3)	73 (10.6)	
Current	n (%)	321 (11.6)	254 (12.2)	67 (9.8)	
eGFR, mL/min/1.73m^2	median (IQR)	86.3 (71.8 - 100.9)	86.5 (71.9 - 101.3)	85.5 (70.9 - 99.2)	0.08
≥90	n (%)	1,194 (43.2)	914 (44.0)	280 (40.8)	0.51
60-89	n (%)	1,232 (44.6)	921 (44.4)	311 (45.3)	
30-59	n (%)	212 (7.7)	151 (7.3)	61 (8.9)	
15-29	n (%)	23 (0.8)	16 (0.8)	7 (1.0)	
<15/RRT	n (%)	101 (3.7)	73 (3.5)	28 (4.1)	
Urine PCR, mg/mmol	median (IQR)	8.5 (6 - 13.3)	8.3 (6 - 13.1)	9 (6.4 - 14)	0.03
<15	n (%)	2,118 (79.6)	1,605 (80.2)	513 (77.8)	0.33
15-49	n (%)	408 (15.3)	300 (15.0)	108 (16.4)	
50-99	n (%)	74 (2.8)	50 (2.5)	24 (3.6)	
≥100	n (%)	61 (2.3)	47 (2.3)	14 (2.1)	
Urine ACR, mg/mmol	median (IQR)	0.7 (0.4 - 1.9)	0.7 (0.4 - 1.8)	0.9 (0.4 - 2.2)	0.002
<3	n (%)	2,107 (82.2)	1,605 (83.1)	502 (79.4)	0.07
3 - 29	n (%)	362 (14.1)	262 (13.6)	100 (15.8)	
≥30	n (%)	94 (3.7)	64 (3.3)	30 (4.7)	
APOL1 genotype					0.10
low-risk	n (%)	2,399 (87.6)	1,792 (87.0)	607 (89.4)	
high-risk	n (%)	339 (12.4)	267 (13.0)	72 (10.6)	
Sickle cell trait	n (%)	331 (12.0)	254 (12.3)	77 (11.3)	0.47

Table S2 Characteristics of study participants stratified by *APOL1* and *GSTM1* status

***APOL1* high-risk genotypes**

		Total	*GSTM1* status		p-value
			Inactive	Active	
		N=339	N=267	N=72	
Age, years	mean (sd)	47.9 (9.8)	47.8 (9.9)	48.5 (9.5)	0.58
Sex, female	n (%)	179 (52.8)	141 (52.8)	38 (52.8)	0.99
Region of ancestry					
East Africa	n (%)	8 (2.4)	7 (2.6)	1 (1.4)	0.76
South Africa	n (%)	47 (13.9)	37 (13.9)	10 (13.9)	
Central Africa	n (%)	8 (2.4)	7 (2.6)	1 (1.4)	
West Africa	n (%)	216 (63.7)	172 (64.4)	44 (61.1)	
Caribbean	n (%)	49 (14.5)	37 (13.9)	12 (16.7)	
Other	n (%)	11 (3.2)	7 (2.6)	4 (5.6)	
HIV mode of acquisition					
Heterosexual	n (%)				0.36
MSM	n (%)	273 (80.5)	218 (81.6)	55 (76.4)	
Vertical	n (%)	11 (3.2)	9 (3.4)	2 (2.8)	
Blood products	n (%)	27 (8.0)	21 (7.9)	6 (8.3)	
Unknown	n (%)	3 (0.9)	3 (1.1)	0 (0.0)	
Time since HIV diagnosis, years	mean (sd)	25 (7.4)	16 (6.0)	9 (12.5)	
Previous AIDS	n (%)	84 (25.6)	66 (25.3)	18 (26.9)	0.79
Nadir CD4 cell count, cells/mm^3	median (IQR)	188.0 (72.0-315.0)	190.0 (74.0-305.0)	153.5 (42.0-378.0)	0.70
Recent CD4 cell count, cells/mm^3	median (IQR)	541.0 (384.0-705.0)	550.0 (378.0-709.0)	513.0 (389.0-696.5)	0.45
On antiretroviral therapy	n (%)	332 (97.9)	261 (97.8)	71 (98.6)	0.65
HIV RNA <200 copies/mL	n (%)	312 (92.0)	246 (92.1)	66 (91.7)	0.90
HBsAg positive	n (%)	0	17 (6.4)	5 (6.9)	0.88
Anti-HCV positive	n (%)	4 (1.2)	4 (1.5)	0 (0.0)	0.29
Diabetes	n (%)	29 (8.6)	22 (8.3)	7 (9.7)	0.70
Hypertension	n (%)	170 (50.1)	137 (51.3)	33 (45.8)	0.41

Cardiovascular disease	n (%)	20 (5.9)	13 (4.9)	7 (9.7)	0.12
BMI, kg/m^2					
<18.5	n (%)	0	0	0	0.73
18.5-24.9	n (%)	62 (18.8)	51 (19.6)	11 (15.7)	
25-29.9	n (%)	132 (40.0)	102 (39.2)	30 (42.9)	
≥30	n (%)	136 (41.2)	107 (41.2)	29 (41.4)	
Smoking status					0.50
Never	n (%)	276 (81.4)	219 (82.0)	57 (79.2)	
Ex	n (%)	28 (8.3)	23 (8.6)	5 (6.9)	
Current	n (%)	35 (10.3)	25 (9.4)	10 (13.9)	
eGFR, mL/min/1.73m^2	median (IQR)	74.9 (54.5 - 92.1)	73.3 (54.4 - 91.3)	80.9 (55.9 - 93.1)	0.13
≥90	n (%)	92 (27.1)	69 (25.8)	23 (31.9)	0.83
60-89	n (%)	143 (42.2)	114 (42.7)	29 (40.3)	
30-59	n (%)	41 (12.1)	33 (12.4)	8 (11.1)	
15-29	n (%)	9 (2.7)	8 (3.0)	1 (1.4)	
<15/RRT	n (%)	54 (15.9)	43 (16.1)	11 (15.3)	
Urine PCR, mg/mmol	median (IQR)	9.0 (6.0-14.8)	8.5 (6.0-13.0)	11.5 (7.9-20.0)	0.006
<15	n (%)	220 (77.2)	180 (80.4)	40 (65.6)	
15-49	n (%)	44 (15.4)	29 (12.9)	15 (24.6)	
50-99	n (%)	9 (3.2)	4 (1.8)	5 (8.2)	
≥100	n (%)	12 (4.2)	11 (4.9)	1 (1.6)	
Urine ACR, mg/mmol	median (IQR)	1.0 (0.5-3.1)	0.9 (0.4-2.9)	1.0 (0.5-4.7)	0.33
<3	n (%)	207 (74.2)	165 (75.0)	42 (71.2)	
3 - 29	n (%)	49 (17.6)	38 (17.3)	11 (18.6)	
≥30	n (%)	23 (8.2)	17 (7.7)	6 (10.2)	
Sickle cell trait	n (%)	62 (18.3)	53 (19.9)	9 (12.5)	0.15

***APOL1* low-risk genotypes**

		Total	GSTM1 copies		p-value
			Inactive	Active	
		N=2,399	N=1,792	N=607	
Age, years	mean (sd)	48.0 (10.3)	48.0 (10.4)	48.1 (10.2)	0.83
Sex, female	n (%)	1,386 (57.8%)	1,044 (58.3%)	342 (56.3%)	0.40
Region of ancestry					<0.001
East Africa	n (%)	503 (21.0%)	422 (23.5%)	81 (13.3%)	
South Africa	n (%)	682 (28.4%)	486 (27.1%)	196 (32.3%)	
Central Africa	n (%)	143 (6.0%)	104 (5.8%)	39 (6.4%)	
West Africa	n (%)	620 (25.8%)	442 (24.7%)	178 (29.3%)	
Caribbean	n (%)	289 (12.0%)	209 (11.7%)	80 (13.2%)	
Other	n (%)	162 (6.8%)	129 (7.2%)	33 (5.4%)	
HIV mode of acquisition					0.46
Heterosexual	n (%)	1,960 (81.7%)	1,454 (81.1%)	506 (83.4%)	
MSM	n (%)	58 (2.4%)	49 (2.7%)	9 (1.5%)	
Vertical	n (%)	202 (8.4%)	152 (8.5%)	50 (8.2%)	
Blood products	n (%)	19 (0.8%)	15 (0.8%)	4 (0.7%)	
Unknown	n (%)	160 (6.7%)	122 (6.8%)	38 (6.3%)	
Time since HIV diagnosis, years	mean (sd)	14.1 (6.5)	14.0 (6.6)	14.3 (6.1)	0.39
Previous AIDS	n (%)	552 (23.8%)	404 (23.4%)	148 (24.9%)	0.46
Nadir CD4 cell count, cells/mm^3	median (IQR)	203.0 (83.0-339.0)	205.0 (87.0-341.0)	199.0 (70.0-335.0)	0.32
Recent CD4 cell count, cells/mm^3	median (IQR)	563.0 (402.5-740.0)	562.0 (400.0-734.0)	568.0 (411.0-757.0)	0.60
On antiretroviral therapy	n (%)	2,375 (99.0%)	1,771 (98.8%)	604 (99.5%)	0.15
HIV RNA <200 copies/mL	n (%)	2,237 (93.2%)	1,681 (93.8%)	556 (91.6%)	0.06
HBsAg positive	n (%)	131 (5.5%)	92 (5.2%)	39 (6.5%)	0.24
Anti-HCV positive	n (%)	30 (1.3%)	26 (1.5%)	4 (0.7%)	0.13
Diabetes	n (%)	243 (10.2%)	177 (10.0%)	66 (11.0%)	0.49
Hypertension	n (%)	705 (29.4%)	520 (29.1%)	185 (30.5%)	0.50

Cardiovascular disease	n (%)	0	78 (4.4%)	21 (3.5%)	0.34
BMI, kg/m^2					0.66
<18.5	n (%)	22 (0.9%)	16 (0.9%)	6 (1.0%)	
18.5-24.9	n (%)	541 (22.9%)	415 (23.5%)	126 (21.1%)	
25-29.9	n (%)	844 (35.7%)	628 (35.6%)	216 (36.1%)	
≥30	n (%)	957 (40.5%)	707 (40.0%)	250 (41.8%)	
Smoking status					0.058
Never	n (%)	1,840 (76.7%)	1,356 (75.7%)	484 (79.7%)	
Ex	n (%)	276 (11.5%)	209 (11.7%)	67 (11.0%)	
Current	n (%)	283 (11.8%)	227 (12.7%)	56 (9.2%)	
eGFR, mL/min/1.73m^2	median (IQR)	87.5 (73.9 - 101.4)	88.4 (74.6 - 101.9)	85.9 (71.5 - 99.7)	0.01
≥90	n (%)	1,091 (45.5%)	838 (46.8%)	253 (41.7%)	0.021
60-89	n (%)	1,079 (45.0%)	801 (44.7%)	278 (45.8%)	
30-59	n (%)	169 (7.0%)	116 (6.5%)	53 (8.7%)	
15-29	n (%)	14 (0.6%)	8 (0.4%)	6 (1.0%)	
<15/RRT	n (%)	46 (1.9%)	29 (1.6%)	17 (2.8%)	
Urine PCR, mg/mmol	median (IQR)	8.5 (6.0-13.3)	8.3 (6.0-13.2)	8.8 (6.2-13.8)	0.15
<15	n (%)	1,880 (79.9)	1,413 (80.1)	467 (79.2)	0.88
15-49	n (%)	361 (15.3)	269 (15.3)	92 (15.6)	
50-99	n (%)	65 (2.8)	46 (2.6)	19 (3.2)	
≥100	n (%)	47 (2.0)	35 (2.0)	12 (2.0)	
Urine ACR, mg/mmol	median (IQR)	0.7 (0.4-1.7)	0.7 (0.4-1.6)	0.8 (0.4-2.0)	0.004
<3	n (%)	1,884 (83.2)	1,429 (84.1)	455 (80.4)	0.074
3 - 29	n (%)	310 (13.7)	223 (13.1)	87 (15.4)	
≥30	n (%)	71 (3.1)	47 (2.8)	24 (4.2)	
Sickle cell trait	n (%)	267 (11.2%)	201 (11.3%)	66 (10.9%)	0.80

Appendix

Genetic markers of chronic kidney disease in people of African ancestry with HIV (GEN-AFRICA) Study Group

Barts Health NHS Trust, London (John Booth [PI], Anele Waters, James Hand, Chris Clarke, Sarah Murphy, Maurice Murphy); **Brighton and Sussex University Hospitals, Brighton** (Marion Campbell, Amanda Clarke [PI], Celia Richardson, Alyson Knott, Gemma Weir, Rebecca Cleig, Helena Soviarova, Lisa Barbour, Tanya Adams, Vicky Kennard, Vittorio Trevitt); **Chelsea and Westminster Hospital, London** (Rachael Jones [PI], Jeremy Levy, Alexandra Schoolmeester, Serah Duro; **Guy's and St Thomas' Hospital, London** (Rachel Hilton [PI], Julie Fox, May Rabuya, Lisa Hamzah, Deborah Jordan, Teresa Solano, Hiromi Uzu, Karen Williams, Julianne Lwanga, Linda Ekaette Reid-Amoruso, Hannah Gamlen, Robert J. Stocker, Fiona Ryan, Anele Waters, Karina Mahiouz, Tess Cheetham, Claire Williams, Achyuta Nori, Caroline Thomas, Sivaraj Venkateshwaran, Jessica Doctor, Andrea Berlanga); **King's College Hospital, London** (Frank Post [CI], Beatriz Santana-Suarez, Leigh McQueen, Priya Bhagwandin, Lucy Campbell, Bee Barbini, Emily Wandolo, Tim Appleby, Deborah Jordan, Lois Driver, Sophy Parr, Hongbo Deng, Julie Barber, Andrew Crowe, Chris Taylor, Mary Poulton, Vida Boateng, Marie-Pierre Klein, Caitlin O'Brien, Samuel Ohene-Adomako, Christian Buckingham, Daniel Trotman, Killian Quinn, Kate Flanagan, Verity Sullivan, Holly Middleditch, Itty Samuel, Elizabeth Hamlyn, Candice McDonald, Ana Canoso, Emeka Agbasi, Maria Liskova, Sarah Barber, Amanda Samarawickrama, Zoe Ottaway, Claire Norcross, Amelia Oliveira, Kate Bramham); **Leeds Teaching Hospitals NHS Trust, Leeds** (Jane Minton [PI], Gary Lamont, Ruby Cross, Gaushiya Saiyad, Shadia Ahmed, Rebecca Ashworth, Nicola Window, J Murira, Khine Phyu); **North Manchester General Hospital, Manchester** (Andrew Ustianowski [PI], Gabriella Lindergard, Jonathan Shaw, Sarah Holland, Claire Fox, Jan Flaherty, Margaret-Anne Bevan, Valerie George); **South Tees Hospitals NHS Foundation Trust, Middlesbrough** (David Chadwick [PI], Marie Branch, Pauline Lambert, Adele Craggs); **Mortimer Market Centre, Central and NorthWest London NHS Foundation Trust, London** (Sarah Pett [PI], Hinal Lukha, Nina Vora, Marzia Fiorino, Maria Muller Nunez, Deirdre Sally, James E. Burns, Erica Pool, Rebecca Matthews); **Newcastle upon Tyne Hospitals, Newcastle** (David Ashley Price [PI], Tara Stothard, Bijal Patel, Ian McVittie, Ciara Kennedy, Uli Shwab, Brendan Payne, Sarah Duncan, Jill Dixon, Mathias Schmid, Adam Evans, Christopher Duncan, Ewan Hunter, Yusri Taha, Natasha Astill); **National Cancer Institute, Frederick, USA** (Cheryl Winkler, Elizabeth Binns-Roemer, Victor David); **North Middlesex University Hospital, London** (Jonathan Ainsworth, Rachel Vincent [PI]); **Queen Elizabeth Hospital, Woolwich** (Stephen Kegg [PI], Chloe Saad, Sarah Skinner, Hocine Azzoug, Judith Russell, Tarik Moussaoui, Celia Richardson, Emily Mabonga, Donna Ward, J. Francoise, W. Larbi, Sue Mitchell, A. Manning, V. Russell); **Royal Free London Hospital, London** (Fiona Burns [PI], Mark Harber, Nnenna Ngwu, Jonathan Edwards, Nargis Hemat, Tom Fernandez, Filippo Ferro, Jorge Ferreira, Alice Nightingale, Tasha Oakes-Monger, Darwin Matila, Pedro Nogueira, Victoria Mutagwanya); **St. Georges University Hospitals, London** (Catherine Cosgrove [PI], Lisa Hamzah, Catherine Emily Isitt, Helen Webb, Joyce Popoola, Kate Korley, Mark Mencias, Patricia Ribeiro, Rajeshwar Ramkhelawn, Sandra Oliva Lara, Sara Sajijad); **Imperial College Healthcare NHS Trust, London** (Alan Winston [PI], Jeremy Levy, Amber Shaw, Claire Petersen, Kyle Ring); **University Hospital Lewisham, London** (Melanie Rosenvinge [PI], Chloe Saad, Sarah Skinner, Thembi Moyo, Faith Odong, Katherine Gantert, Tina Ibe); **Africa Advocacy Foundation** (Denis Onyango); **UK CHIC cohort** (Caroline Sabin [PI], Teresa Hill)
